# 4. 137 Hospital Cluster-Randomized Trial of Mupirocin-Chlorhexidine vs Iodophor-Chlorhexidine for Universal Decolonization in Intensive Care Units (ICUs) (Mupirocin Iodophor Swap Out Trial)

**DOI:** 10.1093/ofid/ofab466.004

**Published:** 2021-12-04

**Authors:** Susan S Huang, Edward Septimus, Ken Kleinman, Lauren Heim, Julia Moody, Taliser R Avery, Laura E McLean, Syma Rashid, Katherine Haffenreffer, Lauren Shimelman, Whitney Staub-Juergens, Caren Spencer-Smith, Selsebil Sljivo, Ed Rosen, Russell Poland, Micaela H Coady, Eunice J Blanchard, Kimberly Reddish, Mary K Hayden, Robert A Weinstein, Brandon Carver, Kimberly N Smith, Jason Hickok, Karen Lolans, Nadia Khan, S G Sturdevant, Sujan Reddy, John A Jernigan, John A Jernigan, Kenneth Sands, Jonathan B Perlin, Richard Platt

**Affiliations:** 1 University of California, Irvine, Irvine, CA; 2 Harvard Medical School, Houston, Texas; 3 University of Massachusetts, Amherst, Massachusetts; 4 UC Irvine School of Medicine, Irvine, California; 5 HCA Healthcare, Nashville, Tennessee; 6 Harvard Pilgrim Healthcare Institute, Boston, Massachusetts; 7 Harvard Pilgrim Health Care Institute, Boston, Massachusetts; 8 Massachusetts Bay Transportation Authority, Boston, Massachusetts; 9 Rush University Medical Center, Chicago, Illinois; 10 Ondine, Nashville, Tennessee; 11 Emory University Rollins School of Public Health, Decatur, Georgia; 12 NIH, Baltimore, Maryland; 13 Centers for Disease Control and Prevention, Atlanta, GA

## Abstract

**Background:**

ICU universal decolonization with daily chlorhexidine (CHG) baths plus mupirocin nasal decolonization reduces all-cause bloodstream infections (BSI) and MRSA clinical cultures. We assessed nasal iodophor, an antiseptic less susceptible to resistance, in place of mupirocin.

**Methods:**

We conducted a cluster randomized non-inferiority trial in ICUs, comparing universal decolonization with: 1) **Mupirocin-CHG:** daily CHG baths and 5 days of twice daily nasal mupirocin, to 2) **Iodophor-CHG:** same regimen, substituting twice daily 10% povidone-iodine for mupirocin. All adult ICUs in a hospital were assigned to the same strategy. We compared each hospital’s outcomes during the 18-month intervention (Nov 2017-Apr 2019) to its own baseline (May 2015-Apr 2017), during which all hospitals used mupirocin-CHG. The primary outcome was ICU-attributable *S. aureus* clinical isolates. Secondary outcomes included ICU-attributable MRSA clinical isolates and all-cause BSI. As randomized and as treated analyses used unadjusted proportional hazards models assessing differences in outcomes between baseline and intervention periods across the two groups, accounting for clustering by hospital and patient.

**Results:**

We randomized 137 hospitals with 233 ICUs in 18 states. There were 442,544 admissions in the baseline period and 349,262 in the intervention period. Median ICU length of stay was 4 days. ICU types included mixed medical surgical (56%), medical (9%), surgical (11%), cardiac (15%), and neurologic (9%). CHG adherence was similar in both arms (85%), but adherence was greater for mupirocin (90%) than iodophor (82%). Primary as-randomized results (Table, Figure) exceeded the non-inferiority margin in favor of mupirocin, for *S. aureus* clinical cultures (21% superiority, P< 0.001) and for MRSA clinical cultures (20% superiority, P< 0.001). The regimens had similar BSI hazards. Analyses of fully adherent patients are in progress.

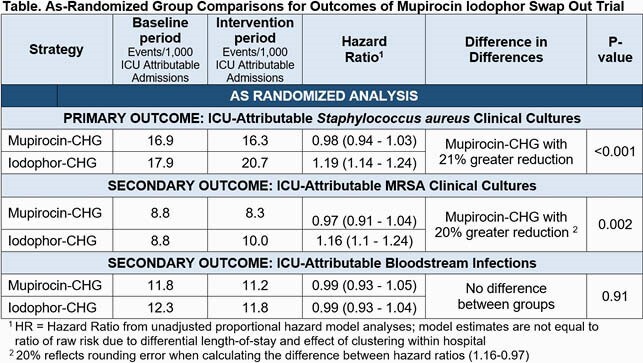

Figure - Primary and Secondary Outcomes of Mupirocin Iodophor Swap Out Trial

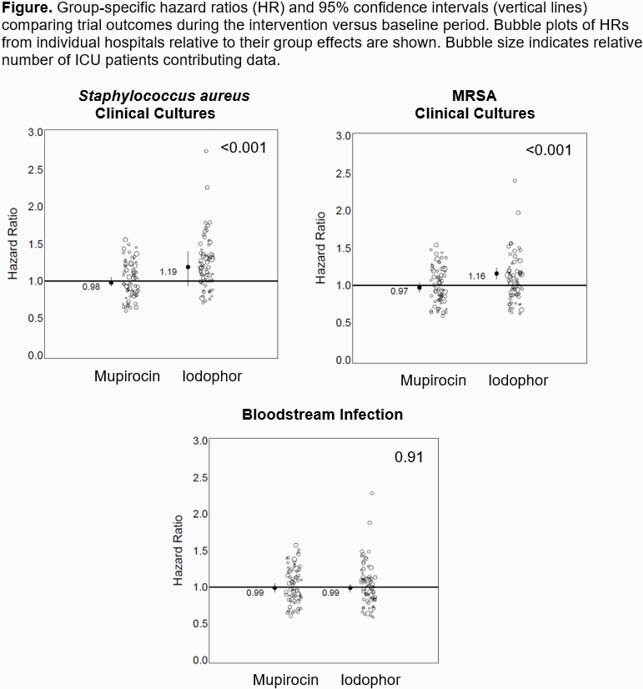

**Conclusion:**

Universal iodophor-CHG was equivalent to mupirocin-CHG for ICU BSI prevention. Mupirocin-CHG was superior to iodophor-CHG for *S. aureus* and MRSA clinical isolates, potentially due to greater adherence to mupirocin.

**Disclosures:**

**Susan S. Huang, MD, MPH**, **Medline** (Other Financial or Material Support, Conducted studies in which participating hospitals and nursing homes received contributed antiseptic and cleaning products)**Molnlycke** (Other Financial or Material Support, Conducted studies in which participating hospitals and nursing homes received contributed antiseptic and cleaning products)**Stryker (Sage**) (Other Financial or Material Support, Conducted studies in which participating hospitals and nursing homes received contributed antiseptic and cleaning products)**Xttrium** (Other Financial or Material Support, Conducted studies in which participating hospitals and nursing homes received contributed antiseptic and cleaning products) **Edward Septimus, MD**, **Medline** (Other Financial or Material Support, Conducted studies in which participating hospitals received contributed antiseptic products)**Molnlycke** (Other Financial or Material Support, Conducted studies in which participating hospitals received contributed antiseptic products) **Ken Kleinman, PhD**, **Medline** (Other Financial or Material Support, Conducted studies in which participating hospitals received contributed antiseptic products)**Molnlycke** (Other Financial or Material Support, Conducted studies in which participating hospitals received contributed antiseptic products) **Lauren Heim, MPH**, **Medline** (Other Financial or Material Support, Conducted clinical trials and studies in which participating hospitals and nursing homes received contributed antiseptic and cleaning products)**Molnlycke** (Other Financial or Material Support, Conducted studies in which participating hospitals received contributed antiseptic product)**Stryker (Sage**) (Other Financial or Material Support, Conducted clinical trials and studies in which participating hospitals and nursing homes received contributed antiseptic product)**Xttrium** (Other Financial or Material Support, Conducted clinical trials and studies in which participating hospitals and nursing homes received contributed antiseptic product) **Julia Moody, MS**, **Medline** (Other Financial or Material Support, Conducted studies in which participating hospitals received contributed antiseptic product)**Molnlycke** (Other Financial or Material Support, Conducted studies in which participating hospitals received contributed antiseptic product) **Taliser R. Avery, MS**, **Medline** (Other Financial or Material Support, Conducted studies in which participating hospitals received contributed antiseptic product)**Molnlycke** (Other Financial or Material Support, Conducted studies in which participating hospitals received contributed antiseptic product) **Syma Rashid, MD**, **Medline** (Other Financial or Material Support, Conducted studies in which participating hospitals received contributed antiseptic product)**Stryker (Sage**) (Other Financial or Material Support, Conducted clinical trials and studies in which participating hospitals and nursing homes received contributed antiseptic product)**Xttrium** (Other Financial or Material Support, Conducted clinical trials and studies in which participating hospitals and nursing homes received contributed antiseptic product) **Katherine Haffenreffer, BS**, **Medline** (Other Financial or Material Support, Conducted studies in which participating hospitals received contributed antiseptic product)**Molnlycke** (Other Financial or Material Support, Conducted studies in which participating hospitals received contributed antiseptic product) **Lauren Shimelman, BA**, **Medline** (Other Financial or Material Support, Conducted studies in which participating hospitals received contributed antiseptic product)**Molnlycke** (Other Financial or Material Support, Conducted studies in which participating hospitals received contributed antiseptic product) **Caren Spencer-Smith, MS**, **Medline** (Other Financial or Material Support, Conducted studies in which participating hospitals received contributed antiseptic product)**Molnlycke** (Other Financial or Material Support, Conducted studies in which participating hospitals received contributed antiseptic product) **Selsebil Sljivo, MPH**, **Medline** (Other Financial or Material Support, Conducted studies in which participating hospitals received contributed antiseptic product) **Ed Rosen, BS**, **Medline** (Other Financial or Material Support, Conducted studies in which participating hospitals received contributed antiseptic product) **Russell Poland, PhD**, **Medline** (Other Financial or Material Support, Conducted studies in which participating hospitals received contributed antiseptic product) **Micaela H. Coady, MS**, **Medline** (Other Financial or Material Support, Conducted studies in which participating hospitals received contributed antiseptic product)**Molnlycke** (Other Financial or Material Support, Conducted studies in which participating hospitals received contributed antiseptic product) **Eunice J. Blanchard, MSN RN**, **Medline** (Other Financial or Material Support, Conducted studies in which participating hospitals received contributed antiseptic product) **Kimberly Reddish, DNP**, **Medline** (Other Financial or Material Support, Conducted studies in which participating hospitals received contributed antiseptic product) **Brandon Carver, BA**, **Medline** (Other Financial or Material Support, Conducted studies in which participating hospitals received contributed antiseptic product) **Kimberly N. Smith, MBA**, **Medline** (Other Financial or Material Support, Conducted studies in which participating hospitals received contributed antiseptic product) **Jason Hickok, MBA**, **Medline** (Other Financial or Material Support, Conducted studies in which participating hospitals received contributed antiseptic product)**Molnlycke** (Other Financial or Material Support, Conducted studies in which participating hospitals received contributed antiseptic product) **Karen Lolans, BS**, **Medline** (Research Grant or Support) **Nadia Khan, BS**, **Medline** (Research Grant or Support) **John A. Jernigan, MD, MS**, Nothing to disclose **Kenneth Sands, MD, MPH**, **Medline** (Other Financial or Material Support, Conducted studies in which participating hospitals received contributed antiseptic product) **Jonathan B. Perlin, MD, PhD**, **Medline** (Other Financial or Material Support, Conducted studies in which participating hospitals received contributed antiseptic product)**Molnlycke** (Other Financial or Material Support, Conducted studies in which participating hospitals received contributed antiseptic product) **Richard Platt, MD, MSc**, **Medline** (Research Grant or Support, Other Financial or Material Support, Conducted studies in which participating hospitals received contributed antiseptic product)**Molnlycke** (Other Financial or Material Support, Conducted studies in which participating hospitals received contributed antiseptic product)

